# Food Liking but Not Wanting Decreases after Controlled Intermittent or Continuous Energy Restriction to ≥5% Weight Loss in Women with Overweight/Obesity

**DOI:** 10.3390/nu13010182

**Published:** 2021-01-09

**Authors:** Pauline Oustric, Kristine Beaulieu, Nuno Casanova, Dominic O’Connor, Catherine Gibbons, Mark Hopkins, John Blundell, Graham Finlayson

**Affiliations:** 1 Appetite Control and Energy Balance Research Group, School of Psychology, University of Leeds, Leeds LS2 9JT, UK; k.beaulieu@leeds.ac.uk (K.B.); ps13doc@leeds.ac.uk (D.O.); c.gibbons@leeds.ac.uk (C.G.); j.e.blundell@leeds.ac.uk (J.B.); g.s.finlayson@leeds.ac.uk (G.F.); 2 School of Food Science and Nutrition, University of Leeds, Leeds LS2 9JT, UK; fsncm@leeds.ac.uk (N.C.); M.Hopkins@leeds.ac.uk (M.H.)

**Keywords:** food reward, liking, implicit wanting, weight loss, follow-up

## Abstract

Food reward (i.e., liking and wanting) has been shown to decrease after different types of weight management interventions. However, it is unknown whether specific dietary modalities (continuous (CER) vs. intermittent (IER) energy restriction) have differing effects on liking and implicit wanting after weight loss (WL) and whether these changes are sustained after 1-year of no-contact. Women with overweight or obesity (age 18–55 years) were randomly allocated to controlled-feeding CER (25% daily energy restriction) or IER (alternating ad libitum and 75% energy restriction days). Study visits were conducted at baseline, post-WL (to ≥5% WL within 12 weeks) and 1-year post-WL. The main outcomes were liking and implicit wanting for 4 categories of common food varying in fat and taste assessed by the Leeds Food Preference Questionnaire. Linear mixed models were conducted on the 30 participants achieving ≥5% WL and 15 returners. After an initial WL of −5.1 ± 0.2 kg, after 1-year 2.6 ± 0.5 kg were regained. Liking but not wanting decreased after WL. Food reward after 1-year did not differ from baseline, but the high loss to follow-up prevents generalization. IER and CER did not differ in their effects on food reward during WL or at 1-year follow-up.

## 1. Introduction

Weight loss (WL) through dietary energy restriction is thought to create a compensatory drive to overeat, which could potentially lead to weight regain [1]. Food reward which can be defined by its dual components of liking (pleasure from the food) and implicit wanting (cue-elicited motivation to eat) [2,3,4,5] has also been proposed to increase during WL. This belief is based on evidence from short-term energy restriction, which has been shown to increase hedonic responses to food [6,7]. However, in a systematic review, food reward was shown to decrease after different modes of WL interventions [8]. Interestingly, the dietary interventions from this systematic review did not include implicit wanting (here-on referred to as ‘wanting’). Therefore, it remains to be elucidated whether dietary WL interventions have a different impact on wanting compared to liking. Indeed, liking and wanting are separable components of food reward with distinguishable brain systems [9], that may have independent roles in eating behavior and in characterizing susceptibility to weight gain [10]. Both liking and wanting are strong determinants of what we eat, but the hedonic value (liking) and the incentive motivation (wanting) can separate under specific situations (e.g., exercise, eating disorders) which remain to be studied [3,11].

Duration of dietary energy restriction has been hypothesized to have a differential impact on food reward, with long-term energy restriction (≥4 weeks) decreasing reward while short-term energy restriction (≤1 day) appear to increase it [8,12,13]. This raises the question of whether intermittent energy restriction (IER; repeated patterns of short-term severe energy restriction interspersed with normal feeding) would generate an increase in food reward compared to continuous energy restriction (CER; daily energy reduction). Alternate day fasting, an IER dietary pattern, consists of alternating between days of severe energy restriction (‘fast days’, e.g., 75% energy restriction typically eaten as one meal) and ad libitum ‘feed days’ [14]. In this study, participants in IER alternated days with 75% energy restriction where they could eat a restricted amount of food throughout the day and ad libitum eating days. While the effect of CER on liking has been studied [15,16,17], the comparison of the effect of IER and CER on food reward has never been explored. IER is often proposed as a dietary pattern that may reduce the compensatory increase in perceived appetite (e.g., hunger, fullness) seen with CER [18] but its effect on wanting is unclear [19]. Consequently, it was hypothesized that IER, as a repeated short-term severe energy restriction interspersed with unrestricted energy intake, might increase food reward, which might prevent successful WL.

Moreover, the permanency and relevance of food reward changes after WL need to be examined. It remains to be elucidated whether any dietary-induced changes in food reward occurring with WL remain after 1-year of no-contact when individuals return to their free-living diet. Indeed, heightened hedonic responses (liking, wanting) have been related to overconsumption and weight regain [5], and “hedonic hunger” has been proposed as a major barrier to WL during follow-up [20]. However, few WL studies have conducted long-term follow-up measures of food reward. Anton, et al. [21] showed a decrease in food cravings after a 24-month weight maintenance intervention, while Buscemi, et al. [22] showed a decrease in cravings during 6 months of WL but no significant changes after a 1-year follow-up. To better understand these discrepancies, the characteristics of follow-up interventions need to be taken into consideration.

This study is a secondary analysis focusing specifically on several dimensions of food reward varying in taste and fat and aiming to explore the effect of CER and IER on changes in food reward after ≥5% WL [23] and at a 1-year follow-up. Other appetite control outcomes during WL, including test meal energy intake, postprandial appetite sensations and eating behavior traits, have been reported elsewhere [24]. The originality of this study is based on the use of behavioral measures of liking and wanting for common and salient dimensions of food and the exploration of individual variability. 

## 2. Materials and Methods 

### 2.1. Participants and Procedure

Women with overweight or obesity (BMI 25.0–34.9 kg/m^2^; age 18–55 years) were recruited from the University of Leeds and surrounding area. This randomized controlled trial received approval from the University of Leeds School of Psychology Research Ethics Committee (PSC-238, date: 10 January 2018) and followed the CONSORT guidelines (see Beaulieu, et al. [24]). Details on inclusion criteria and on the design of the intervention are reported in Appendix A. The main outcomes of the present study were food reward (liking and wanting for food dimensions of fat and taste) measured in a standardized hungry state (3 h after a fixed breakfast) prior to an ad libitum test lunch during study visits at baseline, post-WL and at 1-year follow-up. 

Briefly, 46 women were randomized to IER (ad libitum day alternating with 75% energy restriction day with LighterLife (UK) total diet replacement products provided) or CER (25% daily energy restriction with all foods provided) to ≥5% WL or up to 12 weeks. All study visits took place after a 10–12 h overnight fast and after a ‘fast’ day for the IER group to replicate their energy restriction patterns, as the study visits involved a fixed breakfast and an ad libitum lunch 3 h later. Body weight and composition (BodPod, Life Measurement, Inc., Concord, California, USA) were measured in a fasted state.

As part of the intervention, participants met weekly with a dietitian to monitor WL and were provided with pre-portioned food that required minimal preparation. Meal plans were individually tailored based on food preferences and on calculated energy requirements (resting metabolic rate × physical activity level) obtained from indirect calorimetry (GEM, Nutren Technology Ltd, Daresbury, UK) and a physical activity monitor (SenseWear Armband, BodyMedia, Inc., Pittsburgh, PA, USA) worn during the 7 days leading up to the baseline measurements. Participants had to fill a daily checklist on their weekly meal plan booklet, to report whether they had eaten all the food prescribed or not and space to specify if they had consumed extra food. Adherence to the meal plans was based on those checklists. A day was considered as adherent if reported intake did not exceed prescribed energy intake by >75 kcal. Once the participants reached ≥5% WL at a weekly weigh in (or did not lose ≥5% WL but reached 12 weeks), they continued the intervention for a final week (which included free-living measurements not reported here), and then attended the laboratory for a final study visit. 

**Figure 1:**
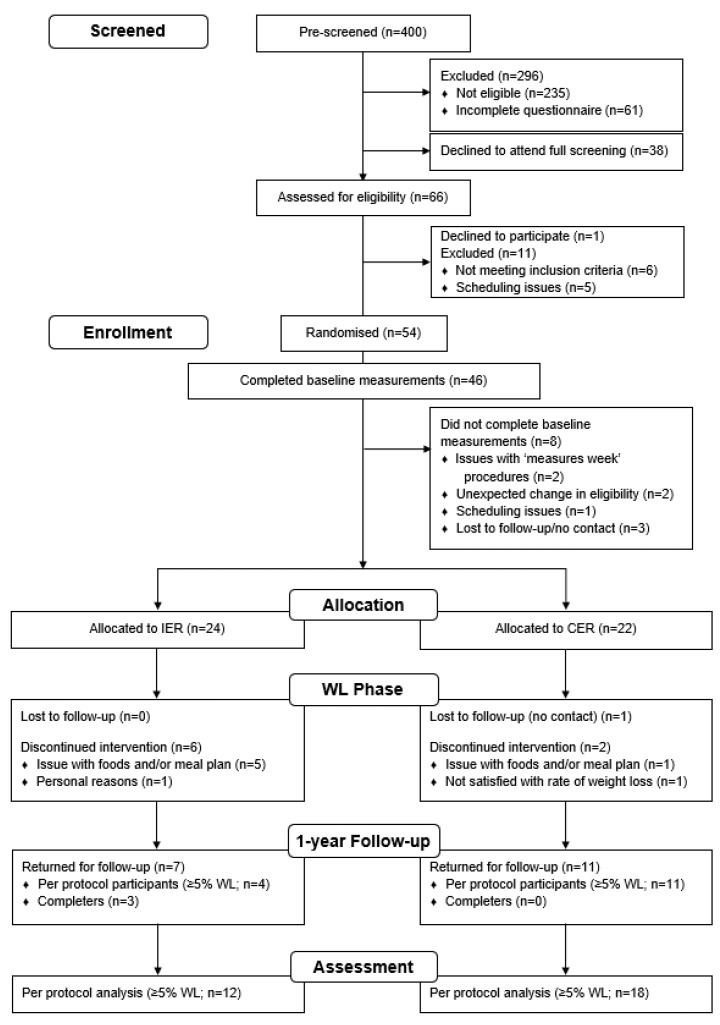
Consort flow-diagram for weight loss and follow-up.

Thirty-seven women completed the intervention and 30 women reached a WL ≥5% within 12 weeks (per protocol). The 37 completers were individually invited four weeks prior to the 1-year date to return for a 1-year follow-up. Participants were not aware of the follow-up measures upon initiation of the WL intervention (clinicaltrials.gov: NCT03447600) but all had consented to be contacted about future studies. No contact was made until invitation to participate in the follow-up study visits, therefore participants did not receive recommendations to pursue their diets after the end of the intervention. Participants were re-screened to confirm eligibility. Figure 1 presents the flowchart of the WL and follow-up study, reporting numbers of individuals invited, consented, lost to follow-up and assessed. 

### 2.2. Food Reward

The Leeds Food Preference Questionnaire (LFPQ) is a validated computerized task [25,26], that has been used in a wide range of research [27,28,29] to measure separate components of food reward. In this study: liking and implicit wanting for 4 categories of food: high-fat-sweet (HFSW), high-fat-savoury (HFSA), low-fat-sweet (LFSW) and low-fat-savoury (LFSA) were reported. Measurements took place three hours after a standardized breakfast individually calibrated to 25% of resting metabolic rate, before an ad libitum lunch (hungry state) and after a ‘fast’ day for the IER group. Pictures of foods (i.e., ready-to-eat foods) were previously selected carefully to be culturally appropriate, correctly recognized, previously consumed, and the categories were matched for palatability and macronutrient content [26]. Food images were individually screened to ensure familiarity and acceptability (see list of foods in Supplementary Materials Table S1).

Implicit wanting was assessed using a forced-choice methodology so that every image from each of the 4 food categories was compared to every other category over 96 trials (food pairs). Participants were instructed to respond as quickly and accurately as they could to indicate which food they most wanted to eat at that moment. Implicit wanting scores were computed for each food category as a function of reaction time weighted by frequency of selection as described in Oustric, et al. [26]. A positive score indicated a more rapid selection for a specific category over the other categories. 

To measure explicit liking, participants rated how much they liked each food (“How pleasant would it be to taste this food now?”) using a 100-point visual analogue scale (VAS). The food images appeared individually on the screen, in a randomized order. The LFPQ has been validated in both laboratory and free-living settings and has been shown to reflect the motivation to eat and pleasure in eating and therefore can affect both the quality and the quantity of food eaten [30,31,32].

### 2.3. Statistics

To analyze the effect of time (baseline, post-WL, follow-up) and diet (CER vs. IER) on liking and wanting for the 4 food categories, linear mixed models were performed on each food reward endpoint. Mixed models were used to take into account the repeated structure of the data and the effect of missing participants between the post-WL and follow-up time points. Factors of time and diet were considered as fixed effects and the participants were entered as a random effect (with random intercepts only). Variable encoding was chosen such that baseline measurement was determined as reference for time and CER as reference for diet. Therefore, post hoc tests were performed to analyze the significance of change between post-WL and follow-up, using the Bonferroni correction. 

As the literature is scarce on the effect of diet modalities on food reward, the simplest model without interactions between diet and time was reported, as the sample size was small. Residuals plots were visually inspected and did not reveal any deviations from linearity, homoscedasticity or normality. Analyses were performed on R [33] using lme4 package [34] to run the mixed models, lmerTest package [35] to obtain *p*-values, and performance package [36] to calculate conditional and marginal R^2^ in order to assess the quality of the model in accordance with Nakagawa and Schielzeth [37]. The full models are reported in Tables S4 and S5 (formula, fixed effect, random effect and goodness of fit measures).

Per protocol analyses (≥5% WL within 12 weeks) included 30 (CER *n* = 18, IER *n* = 12) out of 37 completers and 15 (CER *n* = 11, IER *n* = 4) out of 18 one-year returners. Analyses for the per protocol participants (*n* = 30) are reported. 

## 3. Results

### 3.1. Main Results of the Diet Intervention and Follow-Up

Participant flow chart is presented in Figure 1. Table S2 gives the baseline characteristics of the 30 women with overweight and obesity who reached ≥5% WL. The attrition rate during WL did not differ between groups (CER: 14%, *n* = 19 compared with IER: 25%, *n* = 18, *p* = 0.33), but more completers in CER achieved ≥5% WL within 12 weeks compared with IER (respectively 95%, *n* = 18 and 67%, *n* = 12, *p* = 0.03). Thirty-three % of IER participants (*n* = 4) and 61% of CER participants (*n* = 11) returned for the follow-up in the per protocol analysis (*p* = 0.14).

In terms of WL results and duration of the interventions, 37 women completed the study but did not necessarily achieve ≥5% WL within 12 weeks, with a mean WL of −5.9 ± 1.6% and a range from −8.3% to +0.7% as measured on the final study visit. The 30 per protocol participants lost an average of −6.4 ± 0.9% weight (−5.1 kg), with no difference between diets (CER: 6.3 ± 0.8% (−4.9 kg) in 57 ± 16 days, IER: 6.6 ± 1.1% (−5.3 kg) in 67 ± 13 days) in terms of % WL (*p* = 0.43) or days to reach ≥5% WL (*p* = 0.10).

During follow-up, body weight increased on average by 4.6 ± 5.4% (3.3 kg) ranging from −2.1 to 19.7% in the 15 participants that had achieved ≥5% WL during the intervention. The increase in weight of 19.7% (14 kg) for one participant was considered as an outlier (3.6 SD above the mean) and was removed from the mixed models. Without this outlier, the average change in weight was +3.6 ± 3.6% (2.6 kg) ranging from −2.1 to +8.8%. There was no detectable difference between diets (CER: 3.6 ± 0.9% (2.5 kg), IER: 3.6 ± 1.1% (2.9 kg), *p* = 0.69). Diet adherence measured by the weekly meal plan booklets (during the WL phase only) did not differ between groups (CER: 89.0 ± 9.7%, IER: 81.4 ± 14.6%; *p* = 0.13). Mean calculated daily energy requirement was 2155 ± 399 kcal for CER and 2196 ± 358 kcal for IER (*p* = 0.78). Mean energy prescription was 71.0 ± 4.7% energy requirements for CER (with dietitian adjustments for any WL plateauing) and 24.8 ± 0.3% energy requirements for IER on fast days. 

### 3.2. Changes in Food Reward during WL and Follow-Up

#### 3.2.1. Changes in Liking

**Figure 2:**
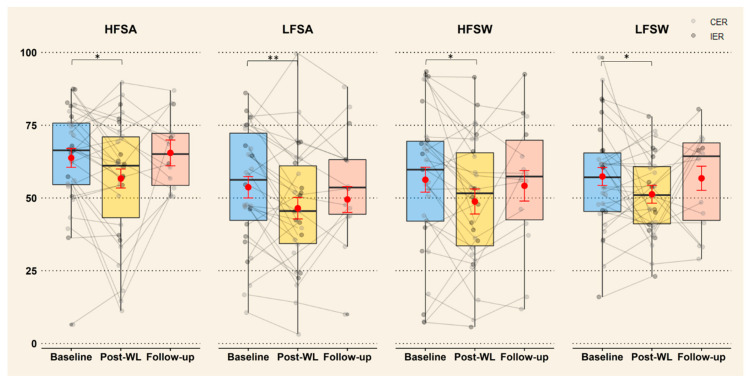
Change in liking for all food categories after ≥5% WL and 1-year follow-up (*n* = 28). The individual data are represented by points (light grey for CER and dark grey for IER) and showed that there was no difference between diets. Estimated means from the mixed models are represented by red points with error bar (SE). Boxplots represent the variability of the data with the median (black line), interquartile range (colored box) and whiskers representing minimum/maximum (Q ± 1.5 x IQR). * Significant changes between baseline, post-WL and follow-up (* *p* < 0.05, ** *p* < 0.01), CER: continuous energy restriction, IER: intermittent energy restriction, HFSW: high-fat-sweet, HFSA: high-fat-savoury, LFSW: low-fat-sweet, LFSA: low-fat-savoury, WL: weight loss.

As shown in Figure 2, mixed models revealed a main effect of time on liking for all food categories with a significant decrease in liking from baseline to post-WL (*p* ≤ 0.05) and no difference between follow-up and baseline values (*p* ≥ 0.25). There was no effect of diet modality (*p* ≥ 0.13). Post hoc tests showed no significant changes in liking for each food category during follow-up (follow-up—post-WL) (*p* ≥ 0.10). However, the estimates of the changes during WL and follow-up were of similar size: HFSA (−7.1 ± 3.2 mm, *p* = 0.03 for WL and 8.8 ± 4.4 mm, *p* = 0.10 for follow-up); LFSA (−7.2 ± 2.6 mm, *p* = 0.01 for WL and 3.0 ± 3.6 mm, *p* = 0.50 for follow-up); HFSW (−7.5 ± 3.1 mm, *p* = 0.02 for WL and 5.4 ± 4.3 mm, *p* = 0.43 for follow-up) and LFSW (−6.1 ± 3.0 mm, *p* = 0.047 for WL and 5.5 ± 4.0 mm, *p* = 0.36 for follow-up).

See Supplementary Materials, Table S3, for the mean value of liking at each time points and changes. Table S4 reports coefficients, SE, *p*-values, CI of the fixed effects with baseline and CER as reference, variance and SD for random effects, and goodness of fit measures.

#### 3.2.2. Changes in Implicit Wanting

**Figure 3:**
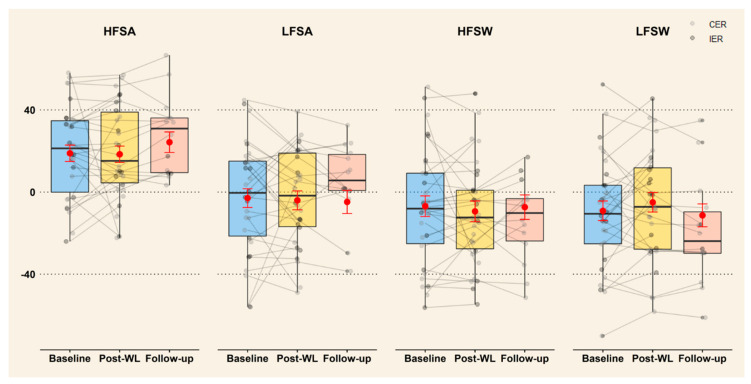
Change in wanting for all food categories after ≥5% WL and 1-year follow-up (*n* = 28). The individual data are represented by points (light grey for CER and dark grey for IER) and showed that there was no difference between diets. Estimated means from the mixed models are represented by red points with error bar (SE). Boxplots represent the variability of the data with the median (black line), interquartile range (colored box) and whiskers representing minimum/maximum (Q ± 1.5 × QR). CER: continuous energy restriction, IER: intermittent energy restriction, HFSW: high-fat-sweet, HFSA: high-fat-savoury, LFSW: low-fat-sweet, LFSA: low-fat-savoury, WL: weight loss.

As shown in Figure 3, mixed models showed no effect of time or diet on wanting for the four food categories (*p* ≥ 0.17). Wanting at follow-up did not differ from baseline and this was the case for each food category (*p* ≥ 0.22). Post hoc analysis showed no significant changes in wanting for each food category during follow-up (follow-up—post-WL) (*p* ≥ 0.38). See Tables S3 and S5 in Supplementary Materials for mean changes, coefficients, SE, *p*-value and CI of the fixed effect and variance, SD for random effect by food category and goodness of fit measures. 

## 4. Discussion

This study aimed to explore (1) the potential effect of different diet modalities (CER vs. IER) on food reward and (2) changes in food reward after ≥5% WL and 1-year follow-up. Contrary to our hypothesis, the modality of WL (CER vs. IER) did not differentially affect food reward although the limited sample size precluded any stronger inferences. Liking but not wanting for food varying in both fat content and sensory properties decreased after ≥5% WL. While scores were numerically comparable to baseline values at the 1-year follow-up, there was a large apparent inter-individual variability and no significant overall change from post-WL to follow-up. A mean weight regain of 3.6% occurred during the 1-year follow-up and improvements in liking observed after ≥5% WL were not maintained. 

### 4.1. Effect of the Modality of WL (CER vs. IER) 

The main finding from this study was that CER and IER did not have a differential impact on liking and wanting. We hypothesized that IER, acting as a form of repeated short-term energy restriction, would increase food reward as seen after a 24-h fast reported in Cameron, et al. [6] and in Thivel, et al. [7]. Both studies measured food reward with the LFPQ in a hungry state (i.e., before lunch) on the day after the fast day, similar to what was conducted in the current study. However, no difference between diet modality was observed for liking or wanting. While this is the first study analyzing the effect of CER and IER on food reward, this finding is in line with other studies reporting no differences in appetite after CER or IER and no compensatory mechanisms after IER [18,38]. Indeed, in this study IER and CER exerted similar effects on food reward and the decrease in liking during WL occurred in a context of improvement in appetite control. The effect of different modalities of energy restriction on liking and wanting remains to be further clarified in future research. 

### 4.2. Changes in Food Reward during WL and Follow-Up

The current outcomes are consistent with the conclusions from our systematic review [8] and showed a decrease in liking for high- and low-energy foods following long-term dietary interventions. This result is in line with other WL studies reporting a decrease in hedonic hunger (measured by the Power of Food Scale), a construct similar to food reward, after a 12-week commercial WL program [39] and a 15-week partial meal replacement intervention [40]. Along the same line, recent reviews reported decreased food craving after long-term energy restriction, supporting a deconditioning model (i.e., uncoupling the association between the craved food and other stimuli) [13,41]. 

On the contrary, one review from Hintze, et al. [42] suggested an increase in liking and wanting following WL. However, in terms of liking, only one longitudinal WL study (8 weeks of caloric deprivation) was reported in which the measure of liking was the participants’ preferred high-energy food, so it could be suggested that frequent exposure to these items had already produced a preference for that food [43]. Moreover, in Cameron, et al. [43] hunger and desire to eat both decreased after the diet-induced WL which seemed contradictory with the increase in liking, so interpretations need to be made with caution. When looking at the studies included in their review, Gilhooly, et al. [44] reported no significant changes in cravings and a decrease in giving in to cravings, and in Jakubowicz, et al. [45], craving increased only in the low-carbohydrate breakfast intervention and decreased in the high-carbohydrate breakfast intervention. Therefore, there seemed to be less evidence for an increase in food reward following WL.

Nevertheless, the decrease in liking during WL was not sustained after 1-year without contact which is consistent with the observed weight regain after returning to a free-living diet. This is in line with Andriessen, et al. [15] showing that liking decreased after diet-induced WL and suggested that this improvement would be altered during the phase of WL maintenance. This is one of very few studies exploring food reward during WL and follow-up but other studies measuring food craving found similar results. Indeed, Buscemi, et al. [22] showed that food cravings decreased during the first 6 months of WL and then did not significantly change during the 1-year follow-up. Interestingly, BMI decreased during WL but increased marginally during follow-up. This result is similar to the current study and the non-significant changes during follow-up could be explained by the high variability in the estimates of food cravings [22] and food reward in the present study. However, due to the high loss to follow-up, other studies are needed to better understand the role of food reward during free-living follow-up.

Another study [21] found that food cravings for high-fat and sweet foods decreased during 2 years of energy restriction diets varying in macronutrient composition, while cravings for fruits and vegetables increased. As participants did regain weight during this study, the sustained decrease in food cravings could be explained by the characteristics of the follow-up in which participants were told to continue their intervention diets. In the current study, participants were not aware of the follow-up study visits and therefore the absence of a supervised follow-up might have weakened the benefit from the supervised WL intervention on their food habits. Therefore, it could be hypothesized that the maintenance of the changes in food reward and food cravings could be due to the maintenance of the “healthier” dietary habits during the WL maintenance. This hypothesis is supported by two other weight management studies [46,47] in which food cravings decreased during WL and then were maintained during weight stabilization. Consequently, in the current study, it can be suggested that the control and restriction over food during the intervention might have contributed, in addition to the personal WL goal, to decrease the liking for food. In contrast, when constraints were removed and participants were free to choose their diet, their habitual conditioned responses to food or food choices may have returned [13]. 

One might ask whether the percentage of WL or rate of WL could have affected the changes in food reward. In this study the percentage of WL was clamped to ≥5% and there was no difference between diets nor between durations to achieve this WL. There were no correlations between percentage of WL or rate of WL (defined as % WL/duration of WL in days) and changes in liking during WL (data not reported here). While the mechanisms of changes in food reward during WL remain to be fully explored in a larger sample, it seems that changes in liking are more related to the consequences of the rigorous dietary intervention rather than the WL per se. 

### 4.3. Separation of Liking and Implicit Wanting

One of the main findings from this study was the decrease in liking but no change in wanting after WL to ≥5%. This is compatible with Berridge’s theory showing that liking and wanting are underpinned by different neural networks [48]. It should also be noted that there was a large apparent inter-individual variability in wanting response to food and therefore a larger sample size may have been required to detect changes. While this was the first diet-induced WL study measuring both liking and wanting, a 12-week exercise training study reported reduced wanting scores for high-fat food, but not liking [49]. It could be hypothesized that exercise affects wanting more than liking, while for diet this might be the contrary. This could be explained by the fact that exercise affects cognitions and executive function while diet modulates eating habits directly [50]. Indeed, during dietary interventions, the relationship with food is externally affected whereas intrinsic motivations held by the individual might not be. On the contrary, during exercise, the strengthening of cognitive processes such as inhibitory control could have a moderating effect on wanting rather than liking [50]. However, it has recently been suggested that chronic exercise could decrease wanting for high-energy food while increasing liking for low-energy food, raising the questions of the mechanisms of change in food reward: cognitive processes, modulation of brain reward systems or other mechanisms [11]. 

One might also ask what is the clinical implication of a decrease in liking, and not wanting, in terms of weight management. Indeed, wanting has been shown to play a larger role than liking in driving overeating [51,52]. The different roles of liking and wanting in weight management could rely on their mode of operation. Liking operates during the consummatory phase of eating whereas wanting is the anticipatory reward or desire to eat that influences the individual before the initiation of consumption [53]. A 10-year longitudinal study reported that preferences for sweet food was a predictor of weight gain among Japanese adult women [54] and preferences for fat have also been related to fat mass or weight gain in populations with obesity [52,55,56,57]. A recent cognitive behavioural therapy induced WL study resulted in altered hedonic but not sensory components of sweet taste, and also suggested that the decrease in palatability might be associated with leptin [58]. Therefore, liking seems also to play a role in weight management and should be explored alongside wanting using both behavioural and neuroimaging methods. On the other hand, as wanting did not change and some weight was regained, it could be suggested that changes in wanting are necessary for weight maintenance, but our study does not permit the formulation of such a firm conclusion.

### 4.4. Limitations and Future Perspectives

There are some limitations in the current study that need to be acknowledged, the first one being the high drop-out at 1-year follow-up. This could be explained by the fact that participants were not aware of the follow-up measurements at the end of WL and that no contact was made, and participants reported being no longer available for the last measurements. This loss to follow-up is important and needs to be acknowledged. Therefore, the conclusions made from the follow-up analysis are limited and cannot be generalized to a larger population. However, the fact that the decrease in liking was not maintained during follow-up is consistent with the weakening of other appetite control variables such as eating behaviour traits and appetite ratings that improved during WL (data not reported here; see Beaulieu et al. [24]). Larger sample sizes are needed to conclude on the role of liking and wanting in weight management and weight regain and its mechanisms, and more specifically the effect of CER vs. IER remains to be explored. 

In terms of design, while the CER group was given pre-portioned foods for all days, the IER group was provided with food packs only for the fast days. The fact that the nature of the food was different in terms of familiarity, type, density (LighterLife total diet replacement products vs. typical food from the supermarket) and that the IER group had access to their own food on feed days, could have impacted food reward. However, the CER group was provided the same number of food packs per week to mitigate any exposure effects to these products, the diets were individually monitored and tailored weekly in both groups to take into account food preferences and no difference in food reward was detected between CER and IER. It would have been interesting to collect information about participants’ eating behaviour at the 1-year follow-up to know whether participants continued their respective diet interventions. This would have informed us about the willingness to follow the intervention in a free-living situation.

It should also be acknowledged that the sample size was small and, taking into account the high variability in food reward, larger randomized controlled trials are warranted to confirm the separation of liking and wanting and the role of food reward in weight management. Therefore, the conclusions made from this analysis need to be considered with caution. Indeed, it can be noticed that the variability of the changes during follow-up appeared to be larger than the variability during WL. In addition, the small sample size during WL could explain why linear mixed models revealed that liking and wanting at the 1-year follow-up did not differ from baseline while the changes from post-WL to follow-up were not significant. Indeed, the size of the estimates were similar after WL and at the follow-up, but the sample size was reduced and consequently the variability was greater. As recommended by Atkinson and Batterham [59], further studies with a non-intervention control group are needed to determine whether the apparent inter-individual variability is due to the intervention.

As suggested by Bryant, et al. [60] it remains to be understood whether the WL itself leads to changes in eating behaviour traits and food reward, or whether the changes in eating behaviour traits or food reward cause the WL, or an interaction between the two. The follow-up was not supervised, contrary to other studies, which could explain the discrepancies observed. However, it gave a picture of what might happen in a free-living scenario without any weight management plans post-intervention. Further studies need to compare different types of weight management interventions to analyze their effect on the psychological and behavioural improvements seen with WL.

## 5. Conclusions

This is the first randomized controlled trial analyzing the role of liking and implicit wanting during CER and IER to ≥5% WL in women with overweight and obesity. This study reinforces the findings from our previous systematic review [8], showing a decrease in food reward during WL regardless of dietary modality, and highlighted a new finding—a dissociation between liking and wanting after different dietary interventions. Contrary to our hypothesis, IER did not have a differential impact on food reward compared to CER. Lastly, changes in liking did not remain after 1 year with no contact, but the high loss to follow-up prevents any generalization of findings. Detailed data analysis showed apparent inter-individual variability in food reward variables but was not able to provide information about the mechanism of changes. It could be proposed that dietary strategies to maintain healthy eating habits would help to sustain appetite control after WL and possibly prevent weight regain [61]. Further studies with larger sample sizes are needed to elucidate the role of liking and wanting during WL and weight maintenance.

## Figures and Tables

**Figure A1:**
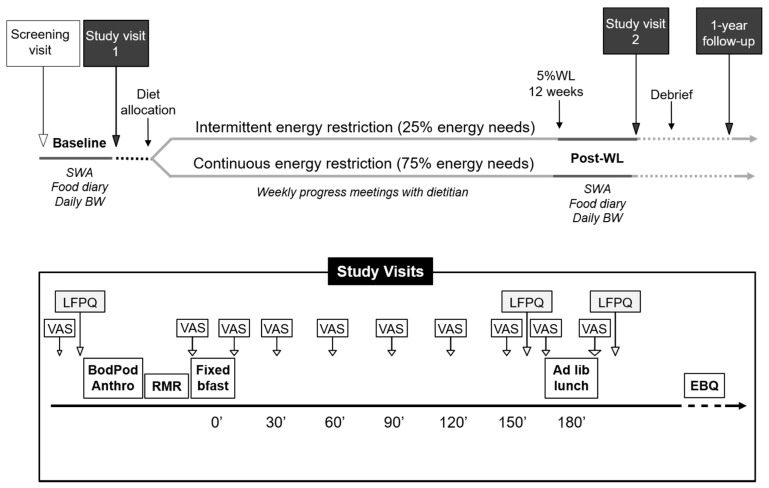
Design of the trial. Anthro: anthropometrics; Bfast: breakfast; BW: body weight; EBQ: eating behaviour questionnaires; LFPQ: Leeds Food Preference Questionnaire; RMR: resting metabolic rate; SWA, SenseWear Armband; VAS: visual analogue scales for appetite ratings; WL: weight loss.

## Data Availability

The data presented in this study are available on request from the corresponding author. The data are not publicly available due to data being still analyzed.

## Supplementary Materials

The following are available online at https://www.mdpi.com/2072-6643/13/1/182/s1, Table S1: List of food used in the LFPQ in this study, Table S2: Baseline characteristics of the 30 women with overweight and obesity who reached ≥5% WL, Table S3: Changes in food reward during weight loss and follow-up, Table S4: Mixed model for liking during weight management, Table S5: Mixed model for implicit wanting during weight management.

## Appendix A

Inclusion Criteria:

- Female participants aged between 18 and 55 years at the time of signing informed consent
- BMI of 25.0–34.9 kg/m^2^

Participants were excluded if:

- they had significant health problems which, in the opinion of the investigator, might jeopardize participant’s safety or compliance with the protocol;
- they were currently enrolled in a weight loss program or following a specific diet plan;
- they had a history of eating disorders;
- they were taking any medication or supplements known to affect appetite or weight within the past month and/or during the study;
- they were pregnant, planning to become pregnant or breastfeeding;
- they had known food allergies or food intolerances (including a history of anaphylaxis to food);
- they were smokers or had recently ceased smoking (<6 months);
- they had lost or gained significant amount of weight in the previous 6 months (±4 kg);
- they exercised >3 days per week or had significantly changed their physical activity patterns in the past 6 months or who intended to change them during the study;
- they were receiving systemic or local treatment likely to interfere with evaluation of the study parameters;
- they worked in appetite or feeding related areas;
- they were shift workers.
