# Supplementary material for: Food Liking but Not Wanting Decreases after Controlled Intermittent or Continuous Energy Restriction to ≥5% Weight Loss in Women with Overweight/Obesity
Source: Nutrients. 2021 Jan 9;13(1):182. doi: 10.3390/nu13010182 (PMC7827400; doi:10.3390/nu13010182)
Supplement: Supplementary file 1 [file nutrients-13-00182-s001.pdf]

Supplementary materials

Article: Food liking but not wanting decreases after controlled intermittent or continuous energy restriction to ≥ 5% weight loss in women with overweight/obesity

Table S1: List of food used in the LFPQ in this study, **Table S2:** Baseline characteristics of the 30 women with overweight and obesity who reached ≥5%WL, **Table S3**: Changes in food reward during weight loss and follow-up, **Table S4:** Mixed model for liking during weight management, **Table S5:** Mixed model for implicit wanting during weight management

Table S1: List of food used in the LFPQ in this study

Food Categories	High-fat	Low-fat
**Savoury**	Cheese	Boiled potatoes
	Peanuts	Pasta in sauce
	Crisps	Rice
	Fries	Bread loaf
**Sweet**	Jam bun	Jelly beans
	Milk chocolate	Popcorn
	Blueberry muffins	Marshmallow
	Donuts	Fruits salad

Table S2: Baseline characteristics of the 30 women with overweight and obesity who reached ≥5%WL

	CER	IER	p-value
**Per protocol (≥5%WL)**	**N = 18**	**N = 12**	
Age (years)	35 ± 9	34 ± 10	0.80
Body mass (kg)	79.2 ± 10.4	81.1 ± 12.2	0.64
Height (cm)	1.65 ± 0.8	1.67 ± 0.9	0.58
BMI (kg/m2)	29.1 ± 2.4	29.1 ± 2.5	0.95
Fat mass (kg)	32.5 ± 8.3	34.0 ± 7.2	0.60
Fat-free mass (kg)	46.7 ± 5.5	47.1 ± 6.6	0.85
Body fat (%)	40.6 ± 6.2	41.7 ± 4.1	0.60
RMR (kcal/day)	1456 ± 214	1441 ± 201	0.85

Means ± SD; BMI: body mass index; CER: continuous energy restriction; IER: intermittent energy restriction; RMR: resting metabolic rate; WL: weight loss. P-values are results of independent sample t-test

Table S3: Changes in food reward during weight loss and follow-up

		Baseline (N = 29)	Post-WL (N = 29)	Follow-up (N = 13)	Changes WL (N = 29)	Change Follow-up (N = 13)
Liking (mm)	HFSA	63.1 ± 17.6	55.9 ± 21.2	64.4 ± 12.1	-7.1 ± 15.4	7.1 ± 21.7
	LFSA	53.2 ± 20.4	46.0 ± 20.9	52.7 ± 19.5	-7.2 ± 15.1	0.3 ± 13.4
	HFSW	55.8 ± 25.7	48.3 ± 21.8	53.1 ± 23.0	-7.5 ± 17.6	8.1 ± 13.3
	LFSW	57.0 ± 19.1	50.9 ± 13.9	54.9 ± 17.1	-6.1 ± 16.8	6.4 ± 17.2
	HFSA	18.9 ± 22.1	18.4 ± 22.0	26.9 ± 19.8	-0.5 ± 20.6	4.3 ± 6.8

Wanting (no unit)	LFSA	-2.6 ± 27.5	-3.7 ± 23.0	3.8 ± 20.0	-1.1 ± 21.1	-3.9 ± 11.3
	HFSW	-7.1 ± 29.1	-9.6 ± 25.5	-13.5 ± 2	-2.5 ± 20.0	2.0 ± 12.5
	LFSW	-9.2 ± 27.2	-5.0 ± 25.9	-17.2 ± 6.1	4.1 ± 18.4	-2.4 ± 5.9

Mean ± SD; HFSW: high-fat-sweet, HFSA: high-fat-savoury, LFSW: low-fat-sweet, LFSA: low-fat-savoury; "Changes-WL" represents changes during WL (post-WL – baseline); "Changes Follow-up" represents changes during follow-up (Follow-up – Post-WL). N = 13 for follow-up as LFPQ data were missing from one participant.

Table S4: Mixed model for liking during weight management

	**Model for HFSA:**	HFSA_PreLunch ~ SV + Condition_5percent + (1 ǀ PPID)						
**Fixed effect**	*Variables*	*Estimate*	*SE*	*t-value*	*df*	*p-value*	*CI_lower*	*CI_higher*
	(Intercept)	59.49	3.95	15.06	44.21	< .001	51.58	67.41
	Post-WL	-7.12	3.25	-2.19	42.78	0.034	-13.64	-0.60
	Follow-up	1.64	4.39	0.37	46.98	0.711	-7.11	10.5
	IER	8.65	5.52	1.56	30.46	0.128	-2.56	19.82
**Random effect**		*Variance*	*SD*					
	PPID	147.5	12.15					
	Residuals	153.2	12.38					
	Number of obs	Groups: 71; PPID: 29						
**Goodness of fit measures**	Conditional R^2: 0.537; Marginal R^2: 0.091; AIC: 605.2; BIC:619.2							
	**Model for LFSA**	LFSA_PreLunch ~ SV + Condition_5percent + (1 ǀ PPID)						
**Fixed effect**	*Variables*	*Estimate*	*SE*	*t-value*	*df*	*p-value*	*CI_lower*	*CI_higher*
	(Intercept)	50.87	4.60	11.07	35.49	< .001	41.62	60.14
	Post-WL	-7.20	2.62	-2.75	42.26	0.009	-12.47	-1.95
	Follow-up	-4.19	3.60	-1.16	44.08	0.251	-11.35	3.12
	IER	5.62	6.81	0.82	29.59	0.416	-8.19	19.41
**Random effect**		*Variance*	*SD*					
	PPID	282.29	16.80					
	Residuals	99.81	9.99					
	Number of obs	Groups: 71; PPID: 29						
**Goodness of fit measures**	Conditional R^2: 0.751; Marginal R^2: 0.046; AIC: 599.9; BIC: 613.5							
	**Model for HFSW**	HFSW_PreLunch ~ SV + Condition_5percent + (1 ǀ PPID)						
**Fixed effect**	*Variables*	*Estimate*	*SE*	*t-value*	*df*	*p-value*	*CI_lower*	*CI_higher*
	(Intercept)	53.20	5.37	9.91	35.47	< .001	42.40	64.04
	Post-WL	-7.48	3.15	-2.37	41.85	0.022	-13.81	-1.16
	Follow-up	-2.07	4.32	-0.48	43.80	0.635	-10.72	6.60
	IER	6.21	7.94	0.78	29.19	0.440	-9.94	22.23
**Random effect**		*Variance*	*SD*					
	PPID	379.3	19.47					
	Residuals	144.2	12.01					
	Number of obs	Groups: 71; PPID: 29						

SV: Study Visits with 3 levels "Baseline" [ref], "Post-WL" and "Follow-up"; Condition_5percent stands for the diets conditions either IER or CER [ref]; (SE): standard error (SD): standard deviation; (df): degree of freedom

**Goodness of fit measures**	Conditional R^2: 0.735; Marginal R^2: 0.038; AIC: 624.2; BIC: 637.8							
	**Model for LFSW**	LFSW_PreLunch ~ SV + Condition_5percent + (1 ǀ PPID)						
**Fixed effect**	*Variables*	*Estimate*	*SE*	*t-value*	*df*	*p-value*	*CI_lower*	*CI_higher*
	(Intercept)	55.30	3.73	14.82	41.93	< .001	47.83	62.81
	Post-WL	-6.12	2.99	-2.05	41.41	0.047	-12.11	-0.13
	Follow-up	-0.60	4.04	-0.15	45.40	0.883	-8.7	7.48
	IER	4.14	5.25	0.79	29.04	0.437	-6.56	14.74
**Random effect**		*Variance*	*SD*					
	PPID	137.2	11.71					
	Residuals	129.3	11.37					
	Number of obs	Groups: 71; PPID: 29						
**Goodness of fit measures**	Conditional R^2: 0.536; Marginal R^2: 0.044; AIC: 595.5; BIC: 609.1							

Table S5: Mixed model for implicit wanting during weight management

	**Model for HFSA**	HFSA_PreLunch ~ SV + Condition_5percent + (1 ǀ PPID)						
**Fixed effect**	*Variables*	*Estimate*	*SE*	*t-value*	*df*	*p-value*	*CI_lower*	*CI_higher*
	(Intercept)	18.99	4.95	3.84	37.20	< .001	9.01	28.93
	Post-WL	-0.50	3.19	-0.16	42.02	0.876	-6.90	5.90
	Follow-up	5.40	4.36	1.24	44.42	0.222	-3.29	14.20
	IER	-0.22	7.23	-0.03	29.44	0.973	-14.82	14.49
**Random effect**		*Variance*	*SD*					
	PPID	302.7	17.40					
	Residuals	147.5	12.15					
	Number of obs	Groups: 71; PPID: 29						
**Goodness of fit measures**	Conditional R^2: 0.676; Marginal R^2: 0.011; AIC: 619.7; BIC: 633.3							
	**Model for LFSA**	LFSA_PreLunch ~ SV + Condition_5percent + (1 ǀ PPID)						
**Fixed effect**	*Variables*	*Estimate*	*SE*	*t-value*	*df*	*p-value*	*CI_lower*	*CI_higher*
	(Intercept)	-1.13	5.68	-0.20	36.02	0.843	-12.58	10.32
	Post-WL	-1.11	3.52	-0.31	41.59	0.755	-8.16	5.95
	Follow-up	-1.88	4.82	-0.39	43.78	0.699	-11.44	7.99
	IER	-3.58	8.35	-0.43	28.97	0.671	-20.51	13.34
**Random effect**		*Variance*	*SD*					
	PPID	411.1	20.27					
	Residuals	179.4	13.40					
	Number of obs	Groups: 71; PPID: 29						
**Goodness of fit measures**	Conditional R^2: 0.698; Marginal R^2: 0.006; AIC: 636.3; BIC: 649.9							
	**Model for HFSW**	HFSW_PreLunch ~ SV + Condition_5percent + (1 ǀ PPID)						

Fixed effect	*Variables*	*Estimate*	*SE*	*t-value*	*df*	*p-value*	*CI_lower*	*CI_higher*
	(Intercept)	-8.31	6.30	-1.32	33.61	0.196	-21.02	4.42
	Post-WL	-2.52	3.35	-0.75	41.31	0.456	-9.25	4.20
	Follow-up	-0.51	4.61	-0.11	42.9	0.913	-9.83	8.67
	IER	2.92	9.40	0.31	28.6	0.759	-16.20	21.94
**Random effect**		*Variance*	*SD*					
	PPID	549.9	23.45					
	Residuals	163.0	12.77					
	Number of obs	Groups: 71; PPID: 29						
**Goodness of fit measures**	Conditional R^2: 0.772; Marginal R^2: 0.005; AIC: 639.2; BIC: 652.8							
	**Model for LFSW**	LFSW_PreLunch ~ SV + Condition_5percent + (1 ∣ PPID)						
**Fixed effect**	*Variables*	*Estimate*	*SE*	*t-value*	*df*	*p-value*	*CI_lower*	*CI_higher*
	(Intercept)	-9.49	6.00	-1.58	33.90	0.123	-21.61	2.61
	Post-WL	4.13	2.97	1.39	42.35	0.171	-1.82	10.08
	Follow-up	-2.22	4.08	-0.54	43.68	0.589	-10.49	5.91
	IER	0.76	9.01	0.08	29.60	0.933	-17.48	19.02
**Random effect**		*Variance*	*SD*					
	PPID	514.6	22.68					
	Residuals	127.8	11.30					
	Number of obs	Groups: 71; PPID: 29						
**Goodness of fit measures**	Conditional R^2: 0.803; Marginal R^2: 0.010; AIC: 626.5; BIC: 640.1							

SV: Study Visits with 3 levels "Baseline" [ref], "Post-WL" and "Follow-up"; Condition_5percent stands for the diets conditions either IER or CER [ref]; (SE): standard error (SD): standard deviation; (df): degree of freedom
